# Supplementary material for: Converting copper sulfide to copper with surface sulfur for electrocatalytic alkyne semi-hydrogenation with water
Source: Nat Commun. 2021 Jun 23;12:3881. doi: 10.1038/s41467-021-24059-y (PMC8222359; doi:10.1038/s41467-021-24059-y)
Supplement: Supplementary file 1 — Supplementary Information [file 41467_2021_24059_MOESM1_ESM.pdf]

Supplementary Information

Converting CuS to Cu with surface sulfur for electrocatalytic semihydrogenation of alkynes with water

Wu et al.

Supplementary Figures

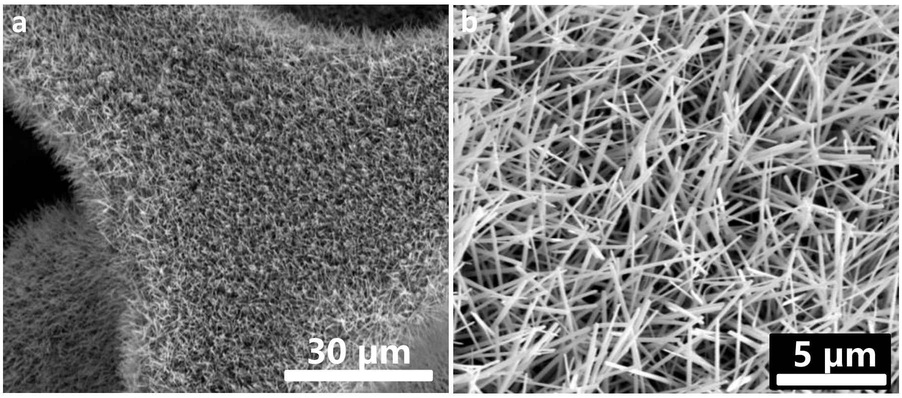

Supplementary Figure 1. SEM images of $Cu(OH)_2$ NAs. **a** Low magnification and **b** high magnification SEM images of the as-prepared $Cu(OH)_2$ NAs with copper foam as substrate.

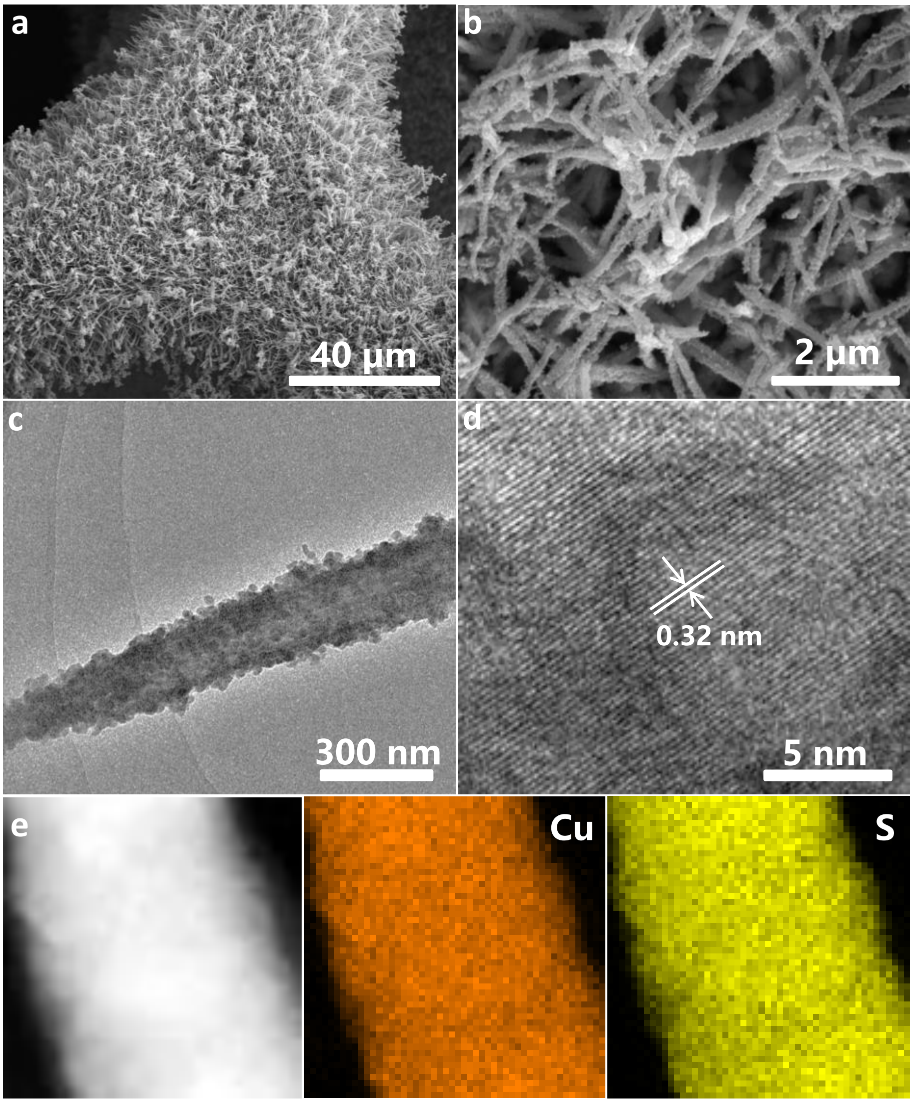

Supplementary Figure 2. TEM and HAADF images of CuS NAs. **a and b** SEM, **c** TEM, **d** HRTEM, and **e** HAADF images, and elemental mapping of the as-prepared CuS NAs.

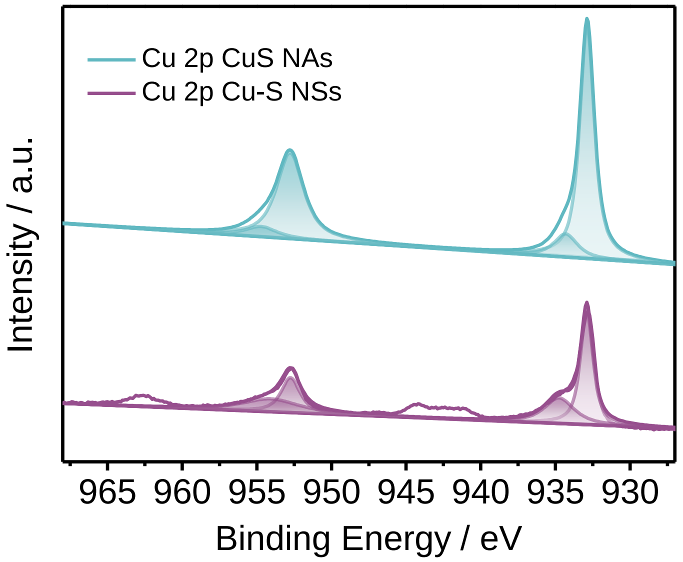

Supplementary Figure 3. XPS spectra of Cu 2p CuS NAs and Cu-S NSs.

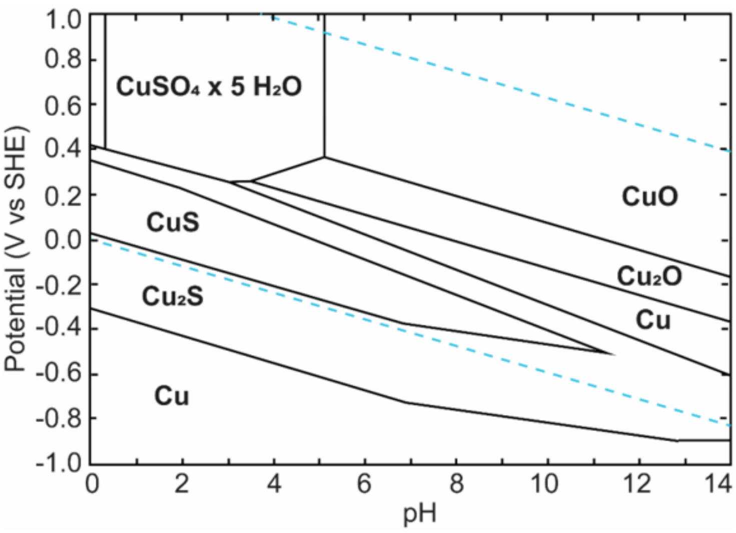

Supplementary Figure 4. Pourbaix diagram of a Cu-S system in H_2O at 25 ℃. The molarities of Cu and S are 1.0 mol/liter H_2O.[1]

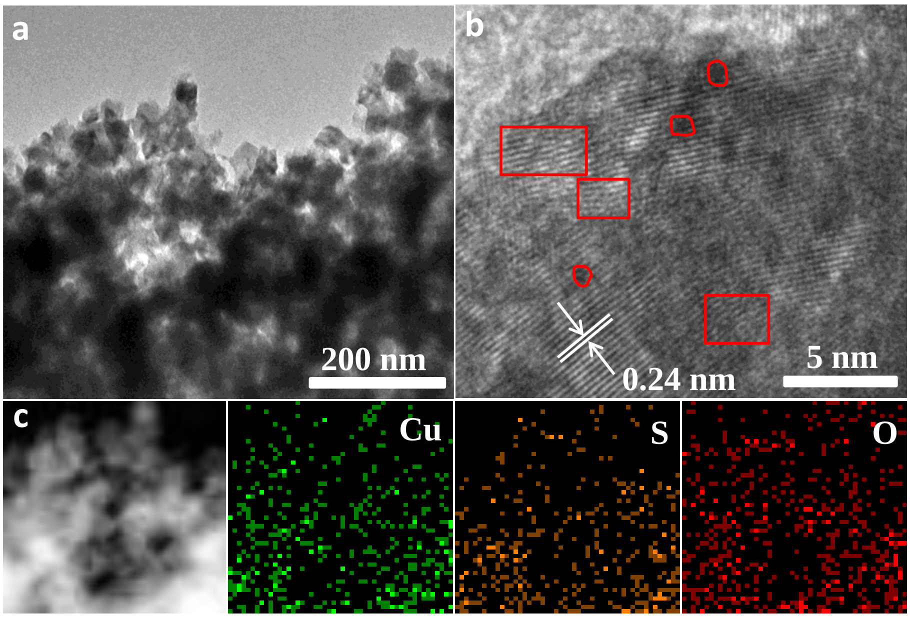

Supplementary Figure 5. TEM and HAADF images of Cu-S NSs. a TEM, **b** HRTEM, and **c** HAADF images, and elemental mapping of the Cu-S NSs.

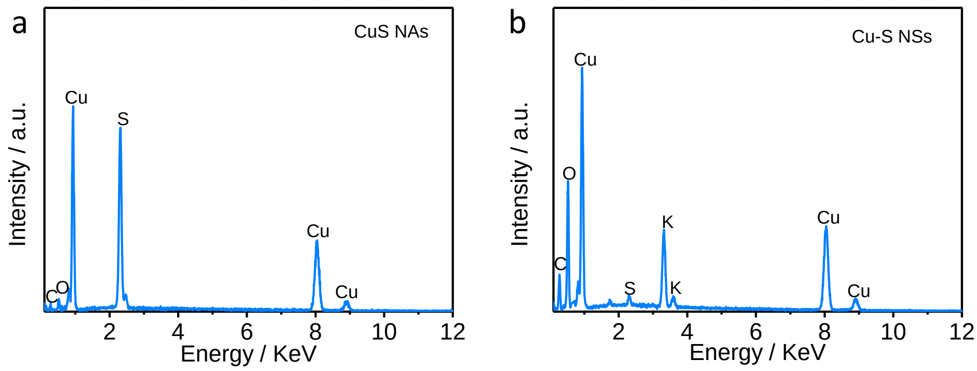

Element	Cu	S	O
Atomic %	49.5	40.5	10.0

Element	Cu	S	O
Atomic %	61.4	5.5	33.1

Supplementary Figure 6. EDS results of CuS NAs and Cu-S NSs.

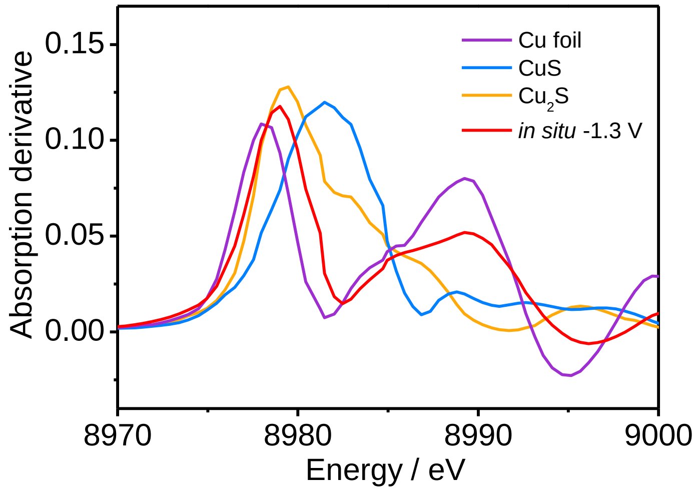

Supplementary Figure 7. First-order derivatives of the XANES spectra for Cu-S NSs at -1.3 V vs. Hg/HgO, Cu foil, CuS, and Cu_2S.

Supplementary Note 1. It is clear that the first-order derivative of the XANES spectra for Cu-S NSs at -1.3 V is dominated by the Cu(0) feature, consistent with the result in Fig. 3, and further proved the successful synthesis of low-coordinated Cu with surface sulfur doping and adsorption.

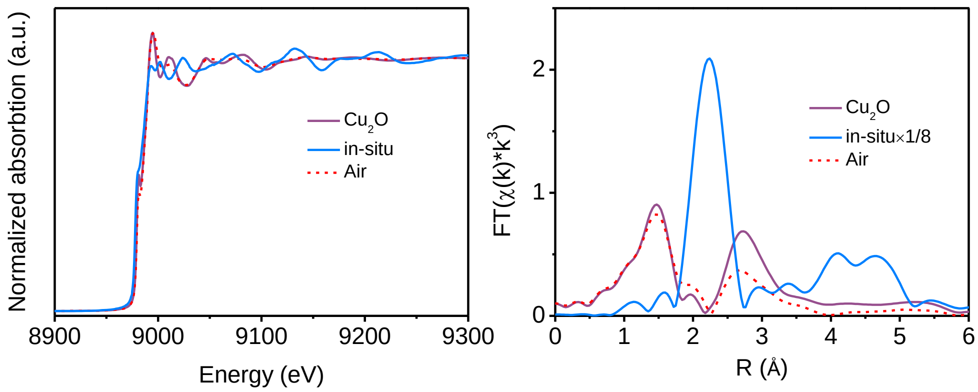

Supplementary Figure 8. XANES and EXAFS spectra of Cu-S NNs exposing to air. **a** XANES and **b** EXAFS spectra of Cu-S NNs exposing to air for 0.5 h.

Supplementary Note 2. After *in situ* electrochemical XAS test, exposing the freshly formed catalyst to air for 0.5 h without bias with following XAS study, the XANES and EXAFS results showed the fresh Cu-S NSs entirely transformed to Cu_2O, verifying Cu_2O from the *ex situ* tests came from the oxidation of Cu-S NSs.

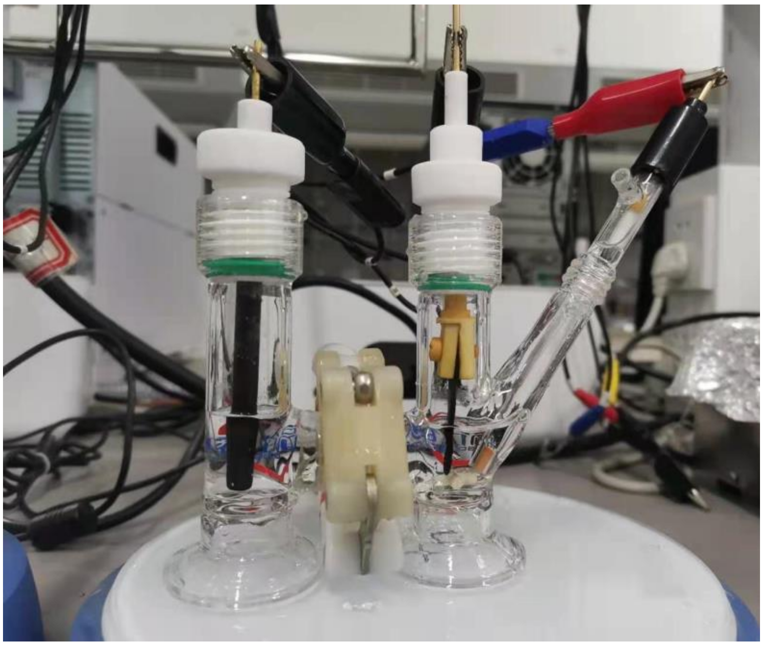

Supplementary Figure 9. Reaction setup for electrochemical semi-hydrogenation of alkynes over Cu-S NSs.

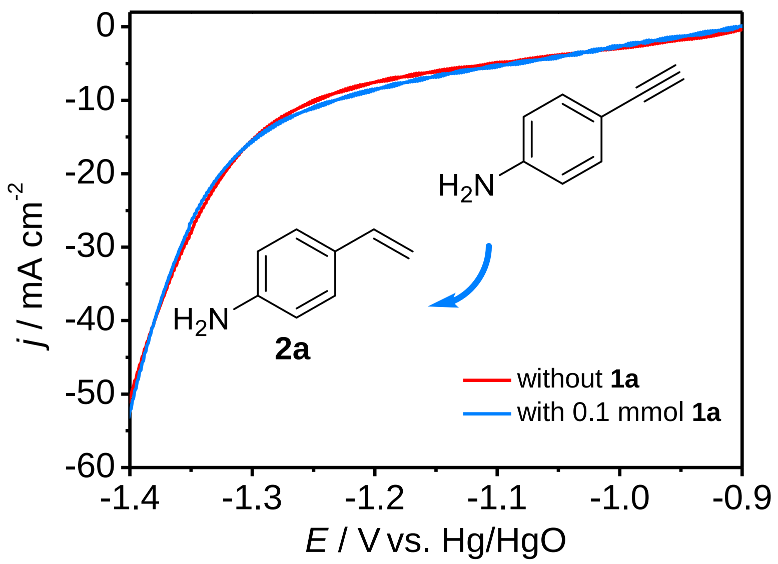

Supplementary Figure 10. LSV curves of Cu-S NSs at a scan rate of 10 mV s^{-1} in 1.0 M KOH solution (Diox/H_2O, 2:5 v/v) with and without 0.2 mmol of **1a**.

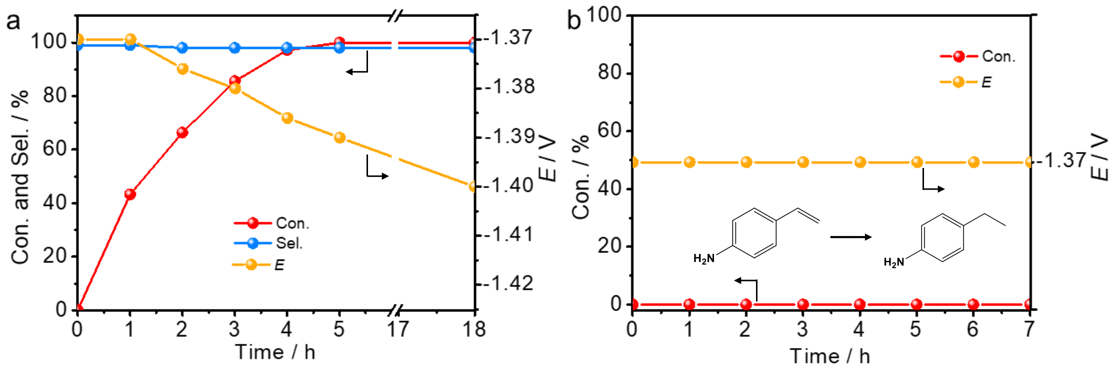

Supplementary Figure 11. a Time-dependent conversion yield (Con.) of 4-ethynylaniline (**1a**), alkene (**2a**) selectivity (Sel.), and potential (E) variations over an in situ formed Cu-S NSs cathode. **b** Time-dependent alkene (**2a**) conversion yield (Con.) and potential (E) variations over an in situ formed Cu-S NSs cathode.

Supplementary Note 3. The long-time electrolysis experiments have been carried under a constant current of -30 mA over the *in situ* formed Cu-S NSs cathode. As shown in Supplementary Figure 11a, the model substrate 4-ethynylaniline (**1a**) could be fully consumed within 5 h to generate the alkene **2a** with 98% selectivity. No selectivity diminish of **2a** was observed even if the reaction time was prolonged to 18 h. This result illustrates that the excellent alkene selectivity is irrelevant to the input potential/current and the reaction time, highlighting the intrinsic control by the Cu-S NSs cathode. In addition, only approximately 30 mV (from -1.37 to -1.4 V) of the potential (*E*) increased during 18 h, which might be due to the hydrogen evolution reaction (HER) that gradually became dominant with alkynes substrate consumption.

To further verify the time-independent selectivity of alkene, alkene **2a** as the starting material was subjected to our reaction system under identical conditions with the reaction of alkyne **1a**. As expected, no alkane product was detected during a 7 h reaction under a constant current of -30 mA. This further rationalized our speculation on Cu-S NSs cathode controlled excellent selectivity of alkene (Supplementary Figure 11b).

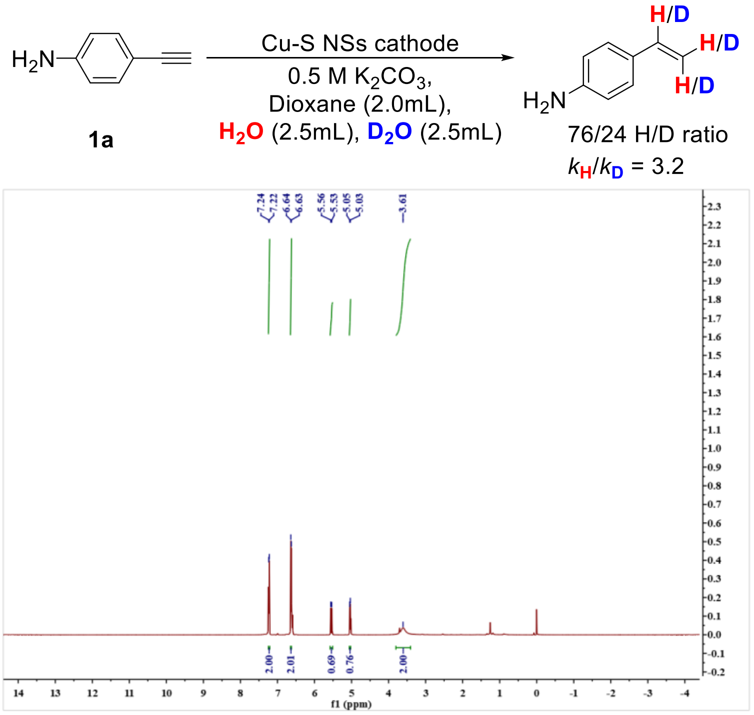

Supplementary Figure 12. Kinetic isotope effect (KIE) experiments.

Supplementary Note 4. The Kinetic isotope effect (KIE) experiments were conducted under the standard conditions at -1.3 V vs. Hg/HgO for 5 h except that 0.5 M K_2CO_3 was used as the electrolyte with using Diox and H_2O/D_2O (1:1) as co-solvents. The H/D ratio of the alkenes product was 3.2 from the ^{1}H NMR data, indicating the dissociation of H_2O was the rate-determining step (RDS) for this electrochemical semi-hydrogenation of alkynes.[2]

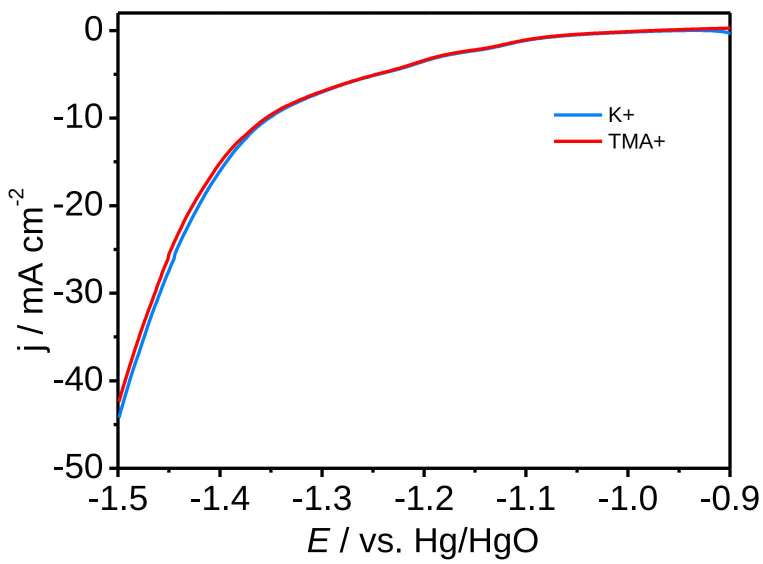

Supplementary Figure 13. LSV curves of Cu NAs in 1.0 M KOH and 1.0 M TMAOH electrolyte for HER.

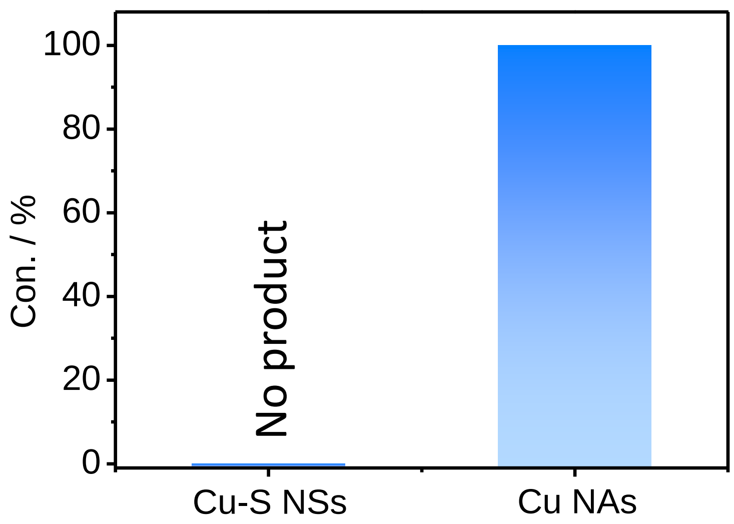

Supplementary Figure 14. Conversion over Cu-S NSs and Cu NAs at -1.4 V vs. Hg/HgO for 4 h, respectively.

Supplementary Note 5. 2a could be completely hydrogenated to the corresponding alkane over $Cu(OH)_2$-derived Cu, but **2a** could not be further hydrogenated to alkane over Cu-S NSs, suggesting the unique advantage of our Cu-S NSs in controlling the high selectivity of alkenes.

^{1}H NMR, ^{13}C NMR spectra, and GC-MS data

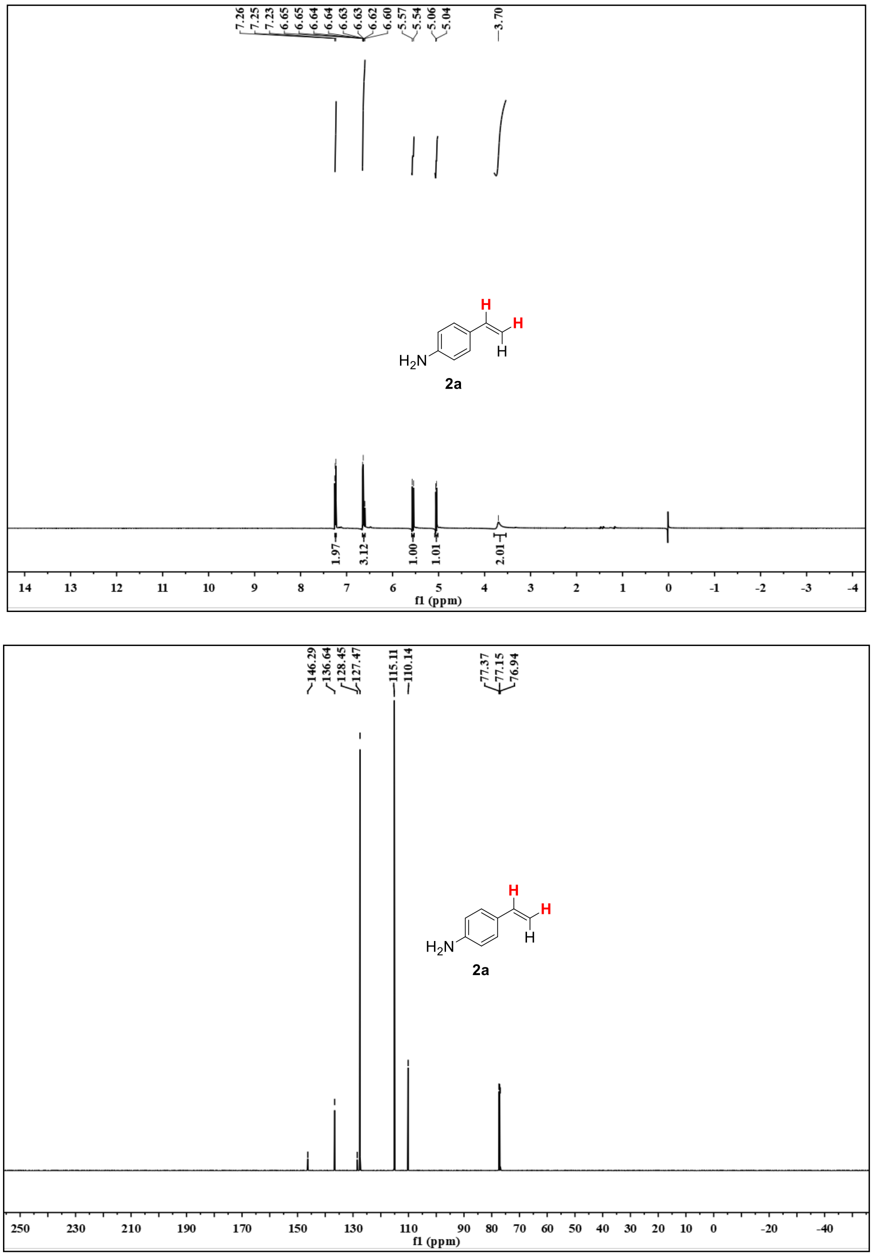

Supplementary Figure S15

^{1}H NMR (600 MHz, $CDCl_3$) δ [ppm] 7.24 (d, J = 8.2 Hz, 2H), 6.65-6.60 (m, 3H), 5.56 (d, J = 17.5 Hz, 1H), 5.05 (d, J =11.2 Hz, 1H), 3.7 (s, 2H); **^{13}C NMR** (101 MHz, $CDCl_3$) δ [ppm] 146.29, 136.64, 133.45, 127.47, 115.11, 110.14; **GC-MS** (EI) 119.1, theoretical value for C_8H_9N [M] 119.1.

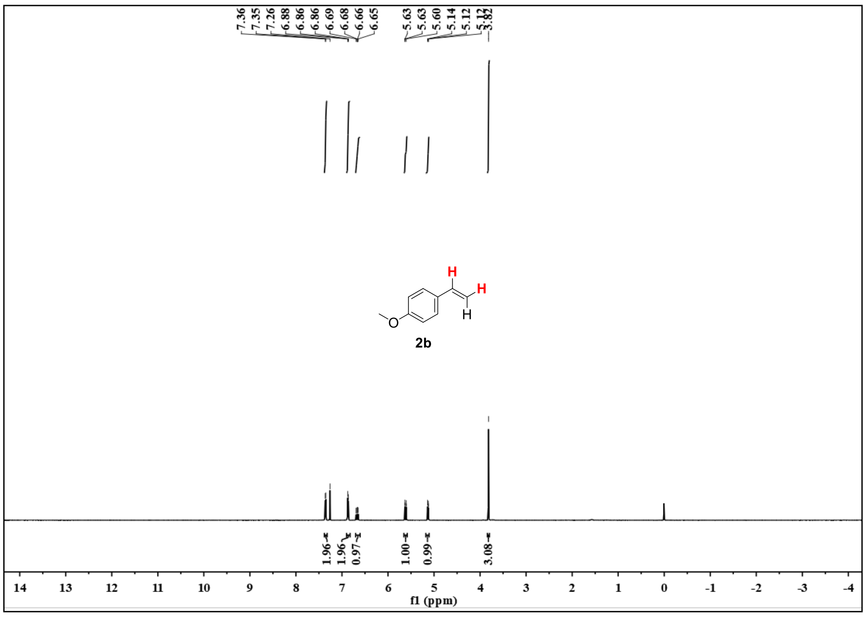

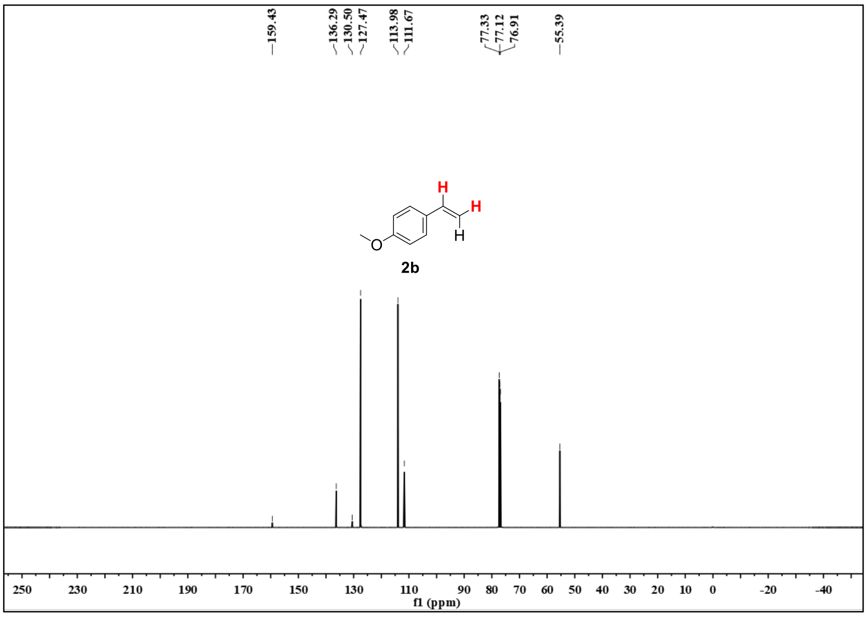

Supplementary Figure S16

^{1}H NMR (600 MHz, $CDCl_3$) δ [ppm] 7.36 (d, J = 9.1 Hz, 2H), 6.87 (d, J = 8.5 Hz, 2H), 6.66 (m, 1H), 5.62 (d, J = 17.7 Hz, 1H), 5.13 (d, J = 10.4 Hz, 1H), 3.82 (s, 3H); **^{13}C NMR** (101 MHz, $CDCl_3$) δ [ppm] 159.43, 136.29, 130.50, 127.47, 113.98, 111.67, 55.39; **GC-MS** (EI) 134.0, theoretical value for $C_9H_{10}O$ [M] 134.1.

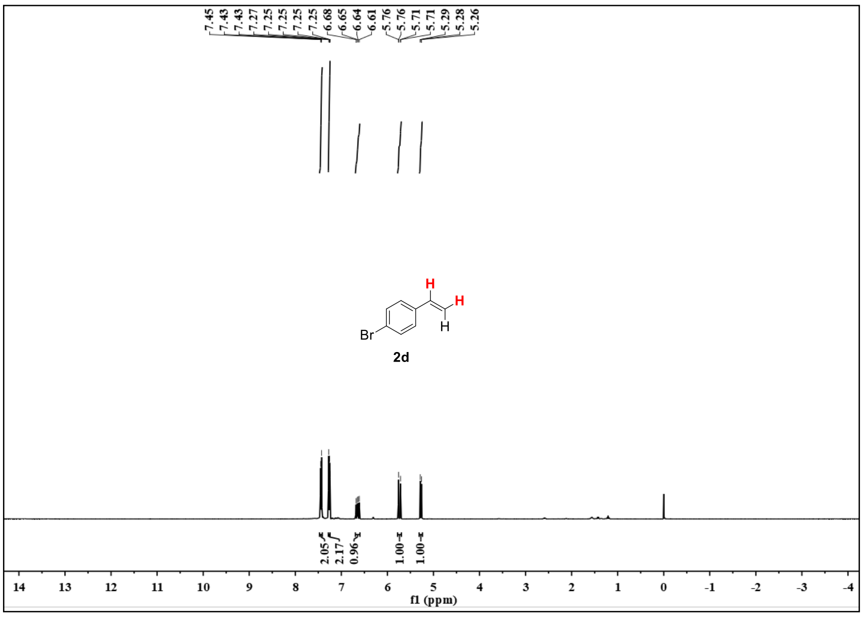

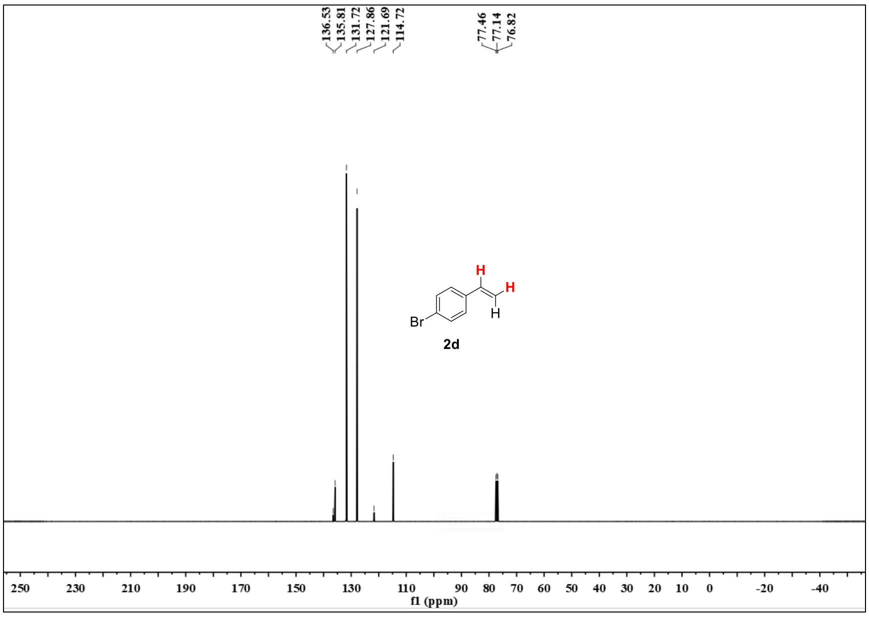

Supplementary Figure S17

^{1}**H NMR** (600 MHz, $CDCl_3$) δ [ppm] 7.44 (d, J = 8.1 Hz, 2H), 7.25 (d, J = 8.9 Hz, 2H), 6.68-6.61(m, 1H), 5.74 (d, J = 17.6 Hz, 1H), 5.27 (d, J = 11.1 Hz, 1H); ^{13}**C NMR** (101 MHz, $CDCl_3$) δ [ppm] 136.53, 135.81, 131.72, 127.86, 121.69, 114.72; **GC-MS** 182.1, theoretical value for C_8H_7Br [M] 181.9.

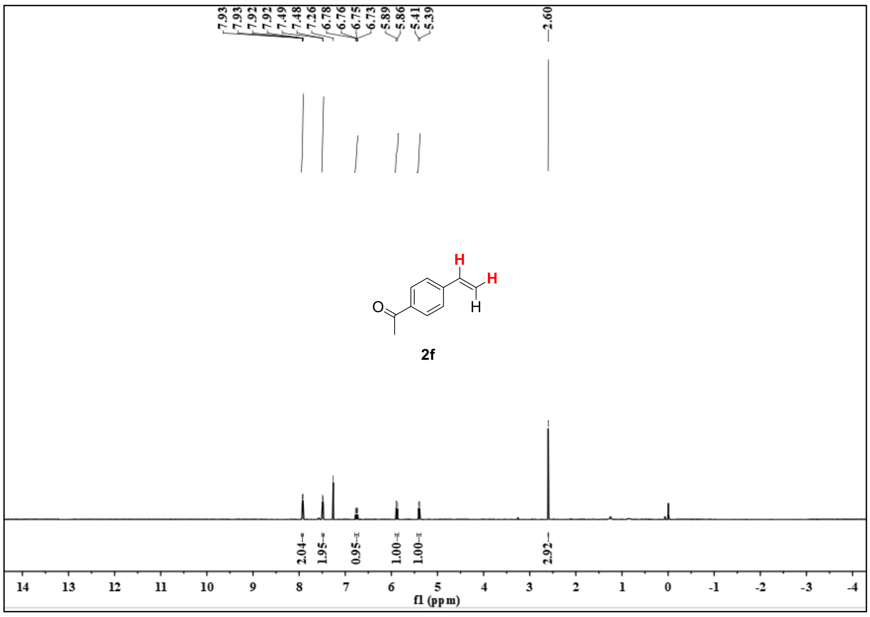

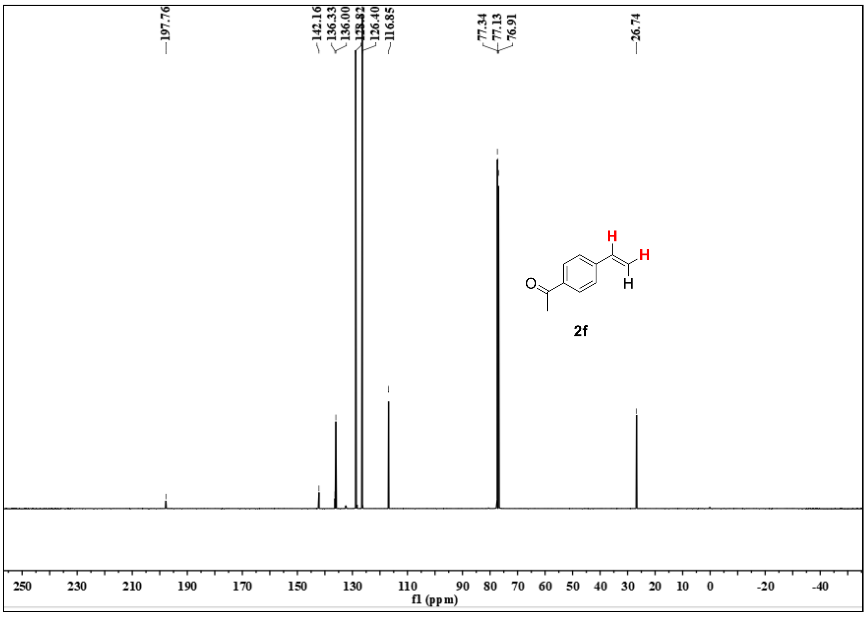

Supplementary Figure S18

^{1}H NMR (600 MHz, $CDCl_3$) δ [ppm] 7.92 (d, J = 8.4, 2H), 7.48 (d, J = 8.3 Hz, 2H), 6.78-6.73 (m, 1H), 5.88 (d, J = 17.5 Hz, 1H), 5.40 (d, J = 11.0 Hz, 1H), 2.60 (s, 3H); **^{13}C NMR** (101 MHz, $CDCl_3$) δ [ppm] 197.76, 142.16, 136.33, 136.00, 128.82, 126.40, 116.85, 26.74. **GC-MS** (EI) 146.1, theoretical value for $C_{10}H_{10}O$ [M] 146.1.

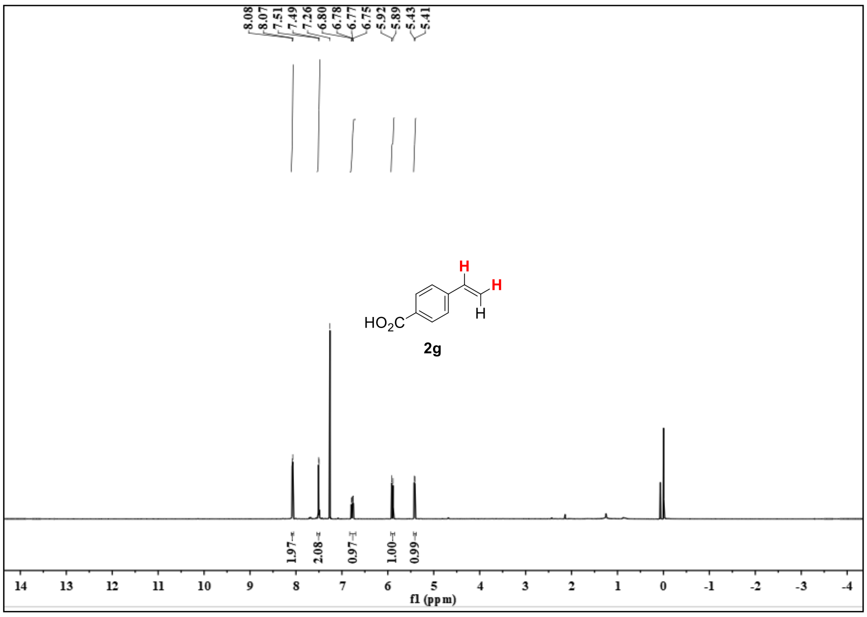

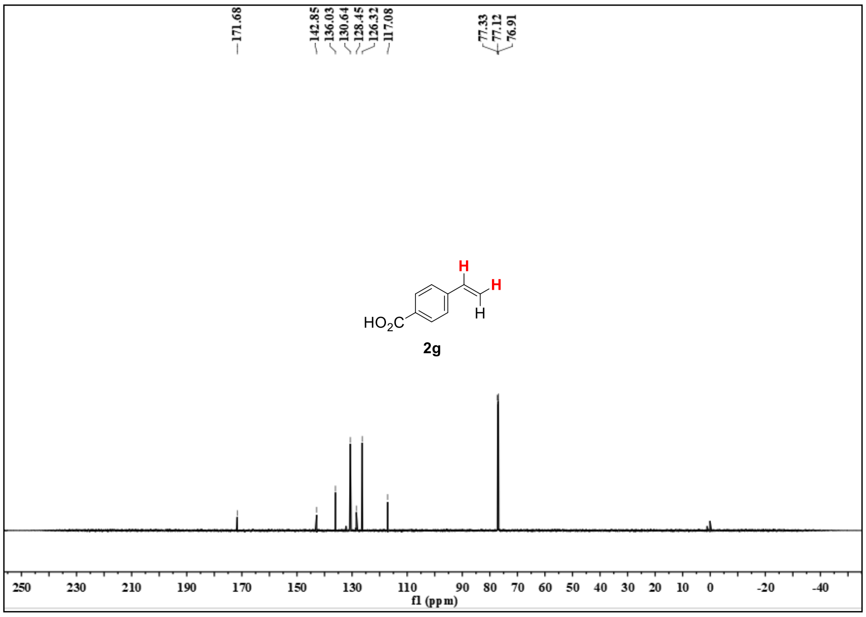

Supplementary Figure S19

^{1}H NMR (600 MHz, $CDCl_3$) δ [ppm] 8.08 (d, J = 8.2 Hz, 2H), 7.50 (d, J = 7.1 Hz, 2H), 6.80-6.75 (m, 1H), 5.90 (d, J = 17.6 Hz, 1H), 5.42 (d, J = 11.4 Hz, 1H); **^{13}C NMR** (101 MHz, $CDCl_3$) δ [ppm] 171.68, 142.85, 136.03, 130.64, 128.45, 126.32, 117.08. **GC-MS** (EI)148.0, theoretical value for $C_9H_8O_2$ [M] 148.1.

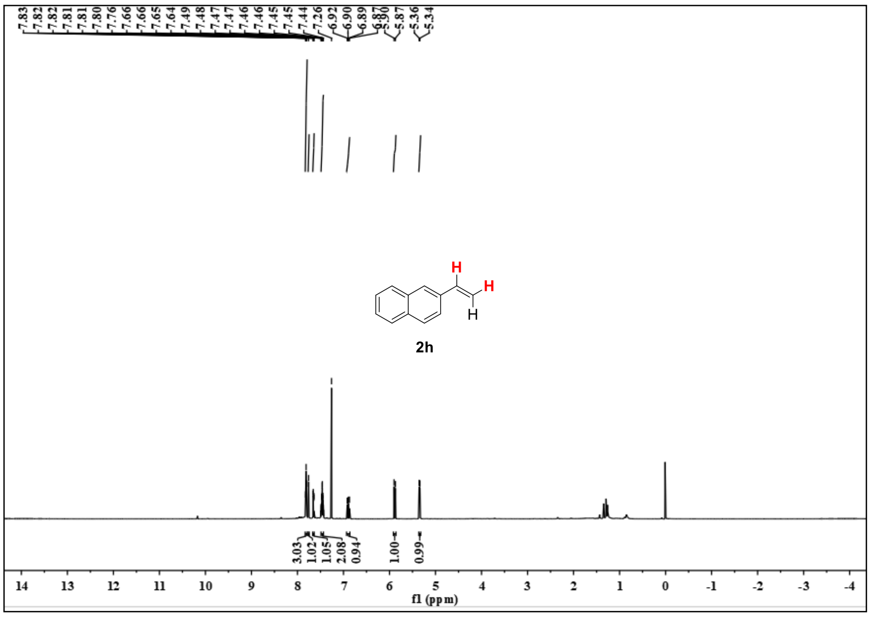

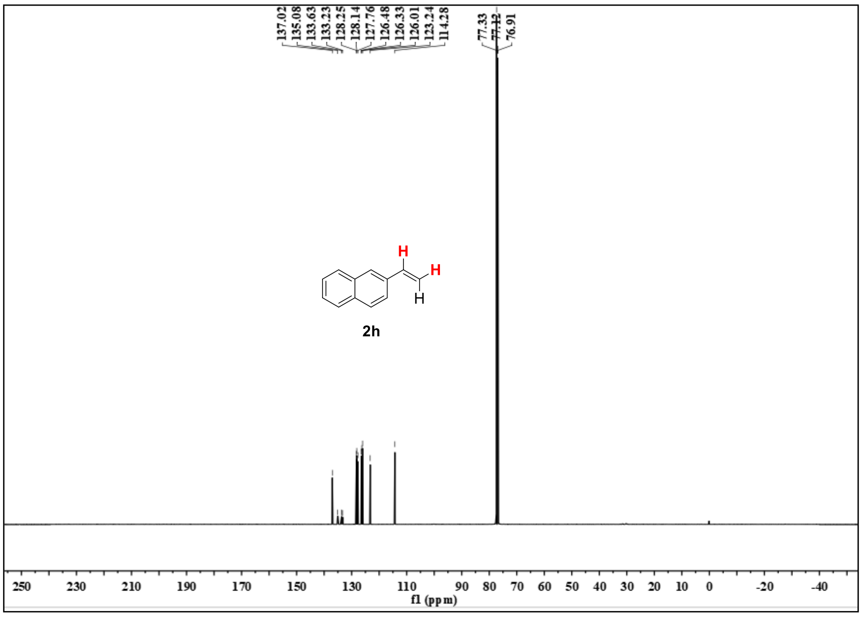

Supplementary Figure S20

[1]**H NMR** (600 MHz, $CDCl_3$) δ [ppm] 7.83 –7.76 (m, 3H), 7.66 (s, 1H), 7.65 (d, J = 8.8 Hz, 1H), 7.49 – 7.44 (m, 2H), 6.92 – 6.87 (m, 1H), 5.88 (d, J = 17.7 Hz, 1H), 5.35 (d, J = 10.8 Hz, 1H); [13]**C NMR** (101 MHz, $CDCl_3$) δ [ppm] 136.02, 135.08, 133.63, 133.23, 128.25, 128.14, 127.76, 126.48, 126.33, 126.01, 123.24, 114.28; **GC-MS** (EI) 154.1, theoretical value for $C_{12}H_{10}$ [M] 154.2.

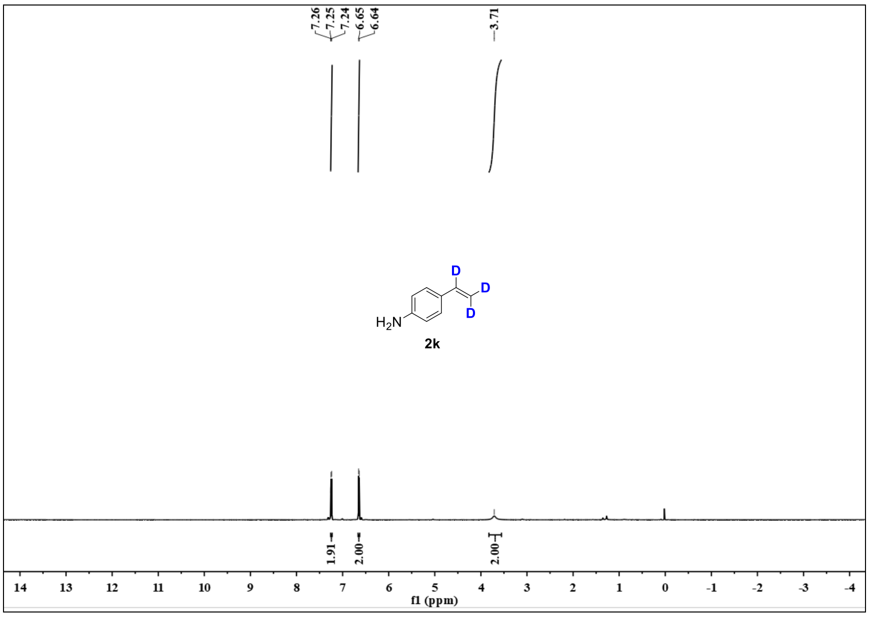

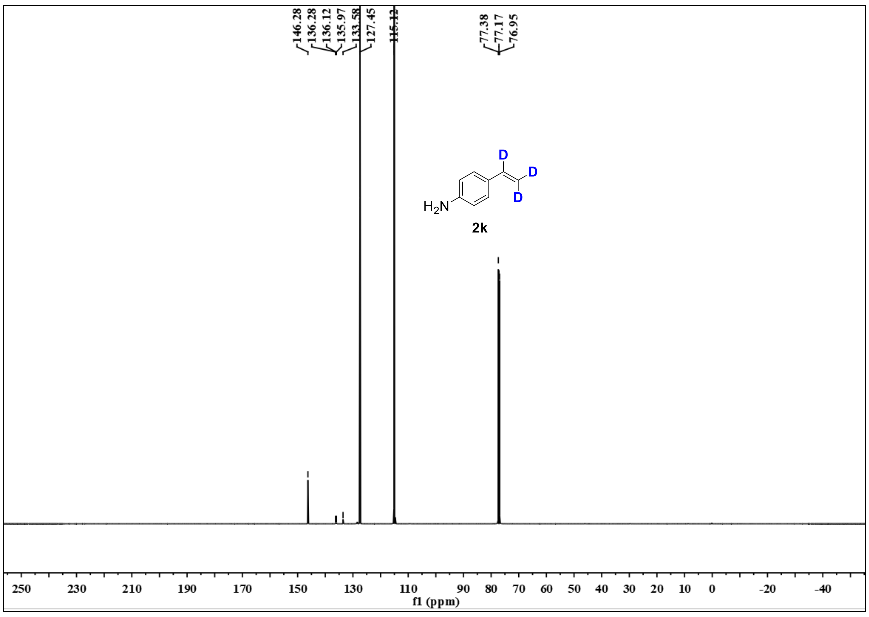

Supplementary Figure S21

Deuterated ratio > 99%, **^{1}H NMR** (600 MHz, $CDCl_3$) δ [ppm] 7.25 (d, J = 7.5 Hz, 2H), 6.64 (d, J = 7.4 Hz, 2H), 3.71 (s, 2H); **^{13}C NMR** (101 MHz, $CDCl_3$) δ [ppm] 146.28, 136.28-135.97 (t, J = 15.7 Hz), 133.58, 127.45, 115.12; **GC-MS** (EI) 122.1, theoretical value for $C_8H_6D_3N$ [M] 122.1.

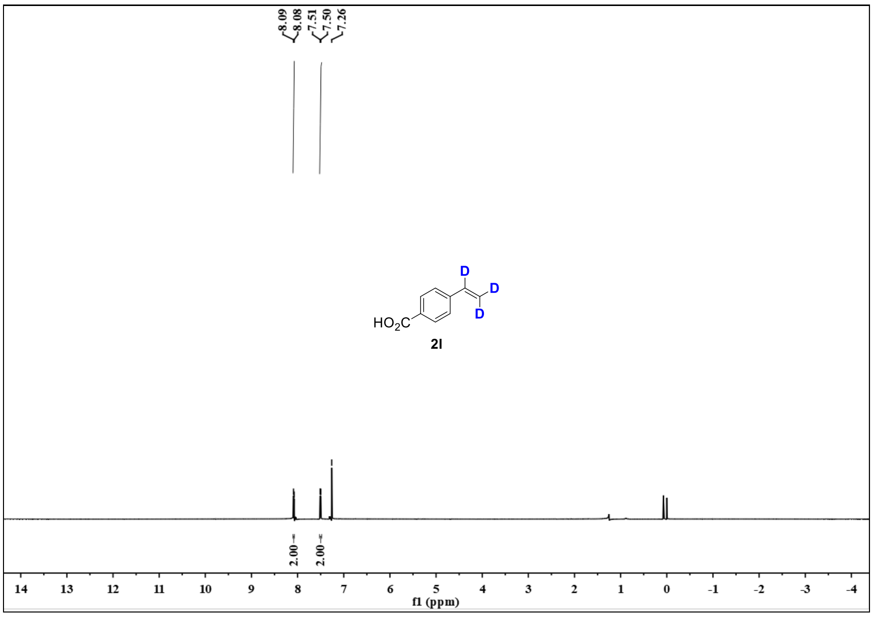

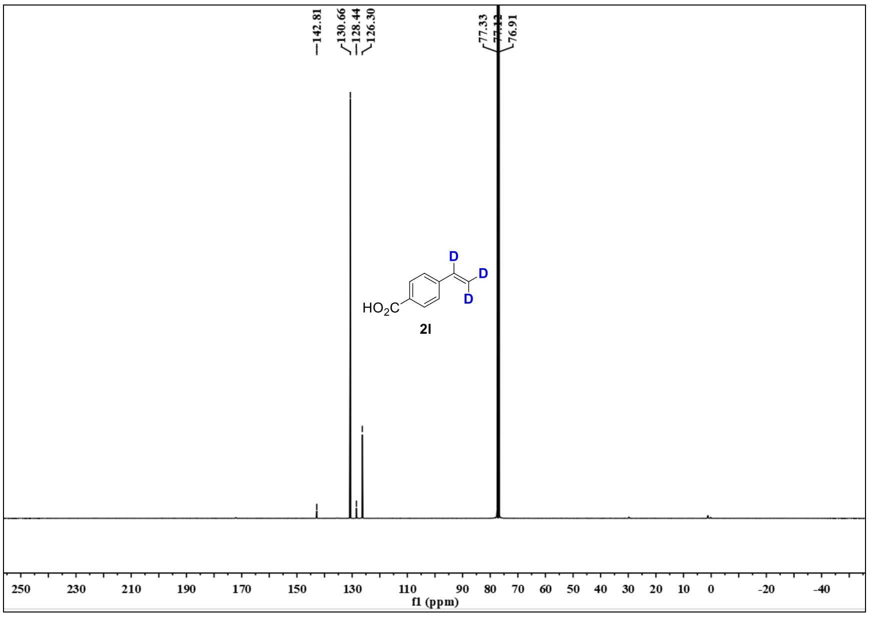

Supplementary Figure S22

Deuterated ratio > 99%, **^{1}H NMR** (600 MHz, $CDCl_3$) δ [ppm] 8.08 (d, J = 8.0 Hz, 2H), 7.50 (d, J = 7.1 Hz, 2H); **^{13}C NMR** (101 MHz, $CDCl_3$) δ [ppm] 142.81, 130.66, 128.44, 126.30; **GC-MS** (EI) 151.1, theoretical value for $C_9H_5D_3O_2$ [M] 151.1.

Supplementary Note 6. Three peaks around 77 ppm in ^{13}C NMR spectra were assigned to the carbon signal of $CDCl_3$. Due to the low vapor pressure or high volatility, parts of the products, including **2c**, **2e**, **2i**, **2j**, were not isolated for the NMR analysis, and their GC-MS data are listed below.

GC-MS of 2c, **2e**, **2i**, **2j:**

2c: GC-MS (EI) 118.0, theoretical value for C_9H_{10} [M] 118.1.

2e: GC-MS (EI) 138.0, theoretical value for C_8H_7Cl [M] 138.0.

2i: GC-MS (EI) 105.1, theoretical value for C_7H_7N [M] 105.1.

2j: GC-MS (EI) 110.0, theoretical value for C_6H_6S [M] 110.1.

Supplementary References

1. Oversteeg, C.H.M., Copper sulfide derived nanoparticles supported on carbon for the electrochemical reduction of carbon dioxide. Catalysis Today DOI: https://doi.org/10.1016/j.cattod.2020.09.020.
2. Liu, K. et al. Cu_2O-Supported Atomically Dispersed Pd Catalysts for Semihydrogenation of Terminal Alkynes: Critical Role of Oxide Supports. *CCS Chem.* **1**, 207-214 (2019).
